# TACKLING THE OPIOID EPIDEMIC: A STATE-WIDE INITIATIVE TO DECREASE OPIOID PRESCRIBING

**DOI:** 10.1093/geroni/igz038.2657

**Published:** 2019-11-08

**Authors:** Jasleen K Chahal, Kimberly Northrip

**Affiliations:** UK HealthCare CECentral, Lexington, Kentucky, United States

## Abstract

More than 500,000 preventable deaths annually have been attributed to the growing opioid epidemic and increased opioid prescribing rates by providers (CDC, 2016). A report by the American Medical Association (AMA) found that only 50% of physicians have taken continuing professional development (CPD) activities on managing pain with opioid alternatives (2016). In 2012, Kentucky passed a comprehensive law regulating the prescribing of controlled substances and requiring ongoing CPD in opioid prescribing and abuse. UK HealthCare CECentral partnered with the Kentucky Office of Drug Control Policy (KODCP) to provide CPD training. This study utilized retrospective learner data collected from CECentral between 2012 – 2017. Since the initiative began in 2012, CECentral has provided this training to 8,893 individuals: 5,877 physicians, 1,527 APNs, and 831 other health professionals. This initiative has resulted in more than 6,000 total participants committing to various forms of clinical practice change to help combat the opioid crisis. Between 2012 and 2016, these commitments resulted in an increase in Buprenorphine/Naloxone prescriptions and prescribers requesting controlled substance reports on their patients. An overall decrease in opioid prescriptions was also shown to be a result of this Kentucky opioid initiative. Results of the study indicate that partnerships between state agencies and CPD providers can lead to practice improvements that address public health concerns. Future research should focus on the efficiency and effectiveness of CPD training and such collaborations as public health initiatives.

